# Teaching Socio-Emotional Competencies Among Primary School Students: Improving Conflict Resolution and Promoting Democratic Co-existence in Schools

**DOI:** 10.3389/fpsyg.2021.659348

**Published:** 2021-06-18

**Authors:** María B. Santamaría-Villar, Raquel Gilar-Corbi, Teresa Pozo-Rico, Juan L. Castejón

**Affiliations:** Department of Developmental and Educational Psychology, University of Alicante, Alicante, Spain

**Keywords:** primary education, socio-emotional skills, school violence, disruptive behaviors, conflict resolution

## Abstract

Teaching socio-emotional skills among primary school students is the key to creating a climate of cooperation in classrooms and reducing disruptive or aggressive behaviors among students. The primary goal of this research is to present an educational proposal for imparting socio-emotional competencies among primary school students. We attempt to impart socio-emotional competencies based on: (1) fostering self-knowledge, self-esteem, and respect for others among students; (2) developing behaviors that allow them to perceive and express feelings and self-regulating emotions; and (3) developing assertive communication skills aimed at improving conflict resolution. This program has been designed in such a way that it is implemented throughout the academic year by organizing bi-monthly sessions of 45 min each, held until the completion of 15 sessions. The sample consists of 100 students in the third grade, with the control and experimental groups having an equal number of students (50 each). The instruments used for this research are: (a) BarOn Emotional Quotient Inventory (Youth Version [BarOn EQ-i:YV]): used for measuring emotional and social functioning; (b) the Matson Evaluation of Social Skills with Youngsters (MESSY): used for assessing social skills; and (c) Questionnaire for the Assessment of School Violence in Preschool and Primary School Questionnaire. To check the effectiveness of the educational intervention, a quasi-experimental design, along with pretest-posttest control group design, is used in accordance with the general linear model. Its effectiveness is also checked using repeated measures analysis of variance. The results show that the program is useful in preventing violent behaviors in the educational field and promoting the development of socio-emotional skills among third grade students. Finally, the applicability of the program to other educational contexts is discussed to enhance students' personal development and decrease the levels of violence found in primary school.

## Introduction

### Theoretical Framework

One of the main objectives of primary education is to train people with knowledge, skills, attitudes, and key competencies for life and personal development. In line with this and notably at the curricular level in this stage, the objective is that students are able to fully mature and achieve happiness, well-being, and maximum academic development. For these reasons, it is important that curriculum designs at this stage provide high school students with the resources and opportunities necessary for their maturation and that give meaning to their academic and personal progression.

It is important to note that the feeling of being appreciated, heard, being part of a community (in this case, educational), and perceiving that personal needs are being addressed ensures that students are better socialized and can manage stress and frustration effectively. It also ensures high levels of well-being and this has a clear impact on the entire school community.

Along these lines, it should be noted that the feeling of satisfaction in the academic environment, both for students and teachers, increases positively when the educational center adopts a learning community dynamic, achieves a positive social climate, records no case of bullying, and members establish bonds of friendship, respect, and positive relationships.

The scientific literature has shown that a child learns to function socially in a school. The peer group, which is one of the main sources of emotional support in childhood, also plays a fundamental role in the development of a student's social competence (Salmivalli et al., 1996; Fekkes et al., 2005; Sharp et al., 2006; Oliver and Candappa, 2007; Riva et al., 2007; Nickerson et al., 2008; Olweus and Limber, 2010; Adolphs, 2013; Oldenburg et al., 2016; Ortega-Ruiz et al., 2016; Santamaría and Valdés, 2017; Arseneault, 2018; Wachs et al., 2019; Pozo-Rico et al., 2020).

The problem arises when violent behaviors are found in this behavior group, which may have detrimental effects on the development, personal adjustment, and academic achievement of students (Barnett et al., 1987; Solberg and Olweus, 2003; Bauman and Del Rio, 2006; Lau and Rosenthal, 2011; Burger et al., 2015; McDougall and Vaillancourt, 2015; Van Noorden et al., 2015; Cuff et al., 2016; Slater and Sanchez-Vives, 2016; Bjereld, 2018). These violent behaviors might also seriously affect the classmate environment (Jolliffe and Farrington, 2006; Salmivalli et al., 2013; Gaffney et al., 2019; Ingram et al., 2019).

In addition, when a student is continuously exposed to such situations of violence, there is a break in the socializing functions performed by peers, thereby forming the sources of stress and school maladjustment (Aceves et al., 2010; Polanin et al., 2012; Veenstra et al., 2014; Brendgen and Troop-Gordon, 2015; Yablon, 2017).

It is possible to identify three types of violent behaviors among peer groups (Coie et al., 1991):

- Reactive violent behavior: occurs in response to the provocation or aggression of the other.
- Instrumental violent behavior: aimed at obtaining an object or a social position.
- Bullying: harassment or mistreatment between equals; the aggression is committed without prior provocation and is directed at a person.

In addition, aggression or violent behaviors (Olweus, 2005) can be classified into the following. (A) Covert aggression: hostility is not directly displayed, but rather, there is irony, jealousy, hatred, yelling, or snorting. (B) Instrumental aggression: this is used as a means to achieve something, rather than causing harm to the victim. It is possible to also differentiate hostile aggression, which is caused by anger and is aimed toward causing pain to someone. (C) Reactive aggression: it occurs as revenge for a previous act. (D) Intimidating aggression: a victim is attacked without prior provocation.

Classmates play a significant role in developing social competence. They are one of the main sources of emotional support in a school and contribute substantially in the formation of our identity (Greene, 2006; Olweus and Limber, 2010; Mulryan-Kyne, 2014; Pincham, 2015; Young-Jones et al., 2015; Gonzalez and Ramirez, 2017; Gilar-Corbi et al., 2020; Saitua-Iribar et al., 2020; Santaolalla et al., 2020; Tyumaseva et al., 2020; Wei et al., 2020). However, when a student is continuously exposed to situations of violence in a school environment, there is a break in the socializing functions provided by peers. In such a situation, they become sources of stress and school maladjustment, leading to school and emotional maladjustment of those who perform and suffer from these violent behaviors (Kochenderfer-Ladd and Skinner, 2002; Bauer et al., 2007; Craig et al., 2009; Ttofi and Farrington, 2011; Chester et al., 2015; Moore et al., 2017; Limber et al., 2018; Gaffney et al., 2019; Jantzer et al., 2019; Smith et al., 2019; Murray and Cousens, 2020; Penalva-Velez et al., 2020).

For these reasons, the research on school violence has increased in the recent decade, constituting a key challenge for schools around the world (Spink, 2005; Court, 2006; Vives, 2014; Becerra et al., 2015; Abu-Nimer and Nasser, 2017; Akyuz et al., 2017; Giavrimis, 2020; Gonzalez et al., 2020; Jurges et al., 2020; Martinez et al., 2020; Ngabirano et al., 2020; Roulston and Cook, 2020; Valero-Valenzuela et al., 2020; Viejo et al., 2020; Wynn et al., 2020).

This school violence includes various types of transgressive behaviors, such as minor criminal acts or more serious behaviors like physical and verbal aggression against teachers and classmates. For this reason, an early and effective educational intervention is very important (Meraviglia et al., 2003; Martin et al., 2005; Mura et al., 2010; Del Rey et al., 2012; Naidoo et al., 2016; Ortega-Ruiz et al., 2016; Yubero et al., 2018; Falla and Ortega-Ruiz, 2019; Vives-Cases et al., 2019; Curtis et al., 2020; Madrid et al., 2020; Peters et al., 2020).

As a result, school violence has negative consequences on all the students involved, while the most damaging effects reverberate on the victim. All the students involved in situations of school violence are at a high risk of suffering from social interaction problems or emotional disorders in the future. The consequences of these on the victim as well as on the aggressor and the observer are pernicious. This is especially so for the victim, who usually suffers from the most negative consequences. They tend to cause school failure and difficulties, high levels of anxiety, dissatisfaction, phobia of going to school, insecurity, negative self-concept, insomnia, eating disorders, depression, aggressive behaviors, and even suicidal attempts (Donoghue et al., 2014; Reuland and Mikami, 2014; Kub and Feldman, 2015; Modin et al., 2015; Duque and Teixido, 2016; Juvonen et al., 2016; Lucia, 2016; Pecjak and Pirc, 2017; Thornberg et al., 2017; Vveinhardt et al., 2017; Juan et al., 2018; Williford and Zinn, 2018; Yang et al., 2018; Garmendia Larranaga et al., 2019; Moyano et al., 2019; Velki, 2019; Li et al., 2020; Nunez-Fadda et al., 2020).

Negative self-concept and low self-esteem will continue to exist in children who have been the victims of school violence until their adult life. They will subsequently favor abuses in their workplaces, family circles, or social spaces (Ma, 2002; del Barrio et al., 2008, 2011; Kartal, 2008; Kartal and Bilgin, 2009; Totura et al., 2009; Pittet et al., 2010; Lopez et al., 2012; Mehta et al., 2013; Twemlow and Sacco, 2013; Cerezo et al., 2015; Duggins et al., 2016; Thornberg et al., 2018; Esposito et al., 2019).

The scientific literature also brings out the negative repercussions of violent behaviors for the aggressor, who learns to achieve his/her objectives improperly. The aggressor tends to reproduce his/her actions through more serious behaviors later in life. In addition, the aggressor tends to be convinced that the rules should not be respected and that the display of aggressive behavior guarantees social popularity (Graham et al., 2006; Gutierrez et al., 2012; Blitz and Lee, 2015; Donoghue and Raia-Hawrylak, 2016; Gonzalez, 2017; Beserra et al., 2019; Angel, 2020; Mendez et al., 2020).

Students who observe violent behaviors in educational settings, even if they do not pick sides in instances of bullying, also manifest negative consequences, such as progressive inhibition while witnessing the pain of others, little empathy and solidarity, feelings of guilt, and isolation, in the future (del Barrio et al., 2008; Simegova, 2009; Lopez et al., 2012; Estevez et al., 2019; Garces-Prettel et al., 2020; Perales et al., 2020).

To summarize, all these aggressive behaviors in the context of schools hinder the normal development of teaching and negatively affect the school environment. As a result, there is a need for novel intervention proposals that are easily transferable to the reality of the classroom and can be adapted to the demands of the 21st century. One such proposal is presented in the current study.

#### Goals, Contents, and Characteristics of the Program for Imparting Socio-Emotional Competencies

The general goal is to promote a social climate of coexistence in the classroom, starting from the promotion of socio-emotional competence among primary education students to ensure that it fosters a healthier relationship among students and enables them to resolve conflicts more peacefully.

The specific goals of the program are detailed below:

- Encourage self-knowledge, self-esteem, and respect for others in students.
- Develop in them behaviors that enable them to perceive and express their feelings and self-regulating emotions.
- Develop their assertive communication skills with the aim of improving conflict resolution.

Basing on these goals, the following contents have been included:

- Content block 1: personal knowledge, self-esteem, empathy, and group cohesion (includes lessons 1–7).
- Content block 2: emotions, emotional regulation, and relaxation (includes lessons 8–11).
- Content block 3: assertiveness, communication, and conflict resolution (includes lessons 12–15).

Note that the training programme adopted two methodologically and conceptually different skills. On the one hand, the skills specifically aimed at vulnerable and at risk of social exclusion schoolchildren (e.g., self-esteem and assertiveness). On the other hand, the skills specially aimed at perpetrators of violence (e.g., self-control, emotional regulation and empathetic behaviors. However, both types of skills have been worked together across the programme, thus strengthening the final goal, that is, conflict resolution and promoting democratic co-existence in schools.

The order of lessons 1–15 is based on a progression from the simplest approach to each of the competencies inherent in training to the acquisition of the most complex ones. Thus, the programme has been designed in such a way that it is implemented throughout the academic year, with bi-monthly sessions of 45 min each held until the completion of the proposed 15 sessions.

The program is deemed to remain flexible in all situations. The sessions can be conducted at any time deemed appropriate, respecting the criteria set forth by teachers. There may be occasions wherein the sessions already worked out can be resumed whenever deemed necessary, or the proposed sessions can be advanced to be carried out at the end of the program in accordance to the occasions that arise within the group.

As a guide, the sessions scheduled for an academic year are provided in Table 1.

**Table 1:** Goals of the program to impart socio-emotional competencies.

**Lesson**	**Goal**	**Lesson's head title**
1	Self-knowledge and self-esteem	“THAT'S HOW I AM”
2	Self-knowledge and self-esteem	“THAT'S HOW WE ARE”
3	Self-knowledge and self-esteem	“STRENGTHS”
4	Self-knowledge and self-esteem	“PERSONAL ACHIEVEMENTS”
5	Self-knowledge and self-esteem	“MY LUGGAGE FOR LIFE”
6	Self-knowledge and self-esteem	“I AM IMPORTANT”
7	Self-knowledge and self-esteem	“WE ARE IMPORTANT”
8	Emotions and emotional regulation	“MY EMOTIONS AND THOSE OF OTHERS”
9	Emotions and emotional regulation	“I EXPRESS EMOTIONS”
10	Emotions and emotional regulation	“I MANAGE MY EMOTIONS”
11	Emotions and emotional regulation	“I RELAX”
12	Assertiveness, communication, and conflict resolution	“I AM ASSERTIVE”
13	Assertiveness, communication, and conflict resolution	“WE COMMUNICATE”
14	Assertiveness, communication, and conflict resolution	“WE COMMUNICATE”
15	Assertiveness, communication, and conflict resolution	“WE SOLVE CONFLICTS”

Finally, the characteristics of the program made it necessary that, before starting the program, it is essential that all the primary education teachers, especially those assigned to conduct classroom sessions, have familiarized themselves with the program and have made this known to the families of students and the entire school community.

For this reason, right at the beginning of the course, primary education teachers receive training from the school's educational guidance team to facilitate the implementation of the program and mobilize necessary resources for its implementation.

Likewise, it is important to explain to the families of students what the program comprises of and offer them guidance on how they could contribute to the reinforcement and consolidation of the skills among students. For this, it would be important to take the advice of the educational guidance team of the school. This would give the teachers the opportunity to meet the families at the beginning of the course and provide them with the resources that are necessary.

This program is not limited to a series of sessions carried out by an isolated teacher in the classroom. The entire educational team is involved in generalizing these learning's during any time of the school day.

Facilitating the generalization of learning is the key that every teacher can take advantage of in occasions that arise spontaneously in the daily life of students. Further, any connection they find in their subjects with what they have worked on the program that week will help them strengthen their remembrance of the lessons learned.

As a result, while it is the teacher who is selected to carry out the proposed sessions with the students, the rest of the teaching team is also involved to ensure the reinforcement and monitoring of the skills acquired.

The contents worked on lesson by lesson will be worked out periodically with the students. In addition, a visual support that helps in remembering the skills is acquired. To do this, a mural with images related to the sessions carried out is created, placing the activities worked on by students on the class board.

## Materials and Methods

### Participants

Two primary schools participated in this research. Both schools have two groups of students for each academic year. Both schools are located in areas with well-off socio-cultural levels. A total of 100 students participated in this study, with 50 students randomly assigned to the experimental condition and the other 50 students assigned as part of the control group. All these students are in the 3rd grade, with the control and experimental groups consisting of similar number of boys and girls (48% male students and 52% female students). The students belonged to 8–9 years age range.

### Instruments

The following criteria were considered while selecting the measurement instruments:

- The conceptual adjustment of the instruments, depending on the variables to be analyzed.
- The reliability and validity of the instruments' psychometric indicators.
- The viability of instruments' application.
- The justification of their adequacy as

Based on these criteria, the following instruments were selected.

#### School Violence in Preschool and Primary School Questionnaire

This questionnaire obtains precise information on different relevant variables such as the type of violence, the places where it occurs, and the frequency of violent behavior (Albaladejo-Blázquez et al., 2013). The questionnaire consists of 30 short and easy-to-understand items. A Likert-type scale with four response options has been used. Given that the questionnaire includes items that assess the frequency with which different situations have been experienced, carried out, or witnessed, the responses to each of the items are: never, few times, many times, and always.

The questionnaire includes four main sections. In the first three sections, we find the assessment of the presence of situations of violence at school from the perspective of the spectator, victim, or the aggressor. The fourth section assesses as to how the subjects react to situations of violence. The factors measured in this study are the following: witnessed violence, lived violence, involving violence, and “what you see”/“what you say.” The psychometric characteristics of this instrument are adequate and show high internal consistency (Cronbach's alpha = 0.86) in the three scales that compose it (violence observed, lived, and carried out). More specifically, the factor “witnessed violence” showed high reliability, with its Cronbach's alpha index being 0.80. In addition, similar reliability was found for the factors “lived school violence” and “realized school violence,” whose Cronbach's alpha indices were 0.71 and 0.79, respectively (Albaladejo-Blázquez et al., 2013).

#### Matson Evaluation of Social Skills With Youngsters

This measure evaluates the degree of adequacy of individuals' social behavior (Matson et al., 1983). The Spanish version of the measure is used in this study (Trianes et al., 2002). The scale makes it possible to evaluate the specific social skills involved in adaptive and non-adaptive social behaviors, considering the students' relationship with their peers and adults. The instrument can be applied to individuals of 4–18 years of age. The instrument has versions of self-report and external evaluation (parents and teachers). The self-report version used in this study has 62 items. The original version has five factors: Aggressiveness/Antisocial Behavior, Appropriate Social Skills, Friendship, Overconfidence/Jealousy/Pride, and Loneliness/Social Anxiety. The Spanish version had the following factors: Aggressiveness/Antisocial Behavior (AAB), Social Skills/Assertiveness (SSA), Conceit/Haughtiness (CH), Loneliness/Social Anxiety (LSA), and MESSY Total Scale. A Likert-type rating scale comprising of 1 (“not at all”) to 5 (“very much”) ratings was used. Recent studies show that the scale has strong psychometric properties, including internal consistency and convergent and divergent validity (Matson et al., 2010). These adequate psychometric properties have also been found in the Spanish version (Méndez et al., 2002).

#### Emotional Quotient Inventory Short EQ-i YV (S)

The Emotional Quotient Inventory is an inventory that covers multiple emotional and social competencies, including an estimate of emotional intelligence as well as a social and affective profile (Bar-On, 1997). The Youth Version (BarOn EQ-i:YV) used in this study assesses the emotional and social functioning of youths aged 7–18, providing an estimate of their underlying emotional and social intelligence (Bar-On and Parker, 2000). It includes 51 items, which are rated on a five-point Likert scale. It evaluates the following general factors of EI: intrapersonal intelligence, interpersonal intelligence, adaptation, and stress management. By adding these dimensions, a general score for Emotional Intelligence is obtained. Higher scores indicate better functioning to meet the demands and challenges of everyday life, while lower scores indicate a greater probability for having emotional, social, and/or behavioral problems. The Spanish version has been used in this study (López-Zafra et al., 2014). All the scales have adequate contrasted validity and the internal consistency of their subscales is between 0.65 and 0.86 (Bar-On, 2004).

Therefore, and in accordance with the scientific literature, MESSY and EQ-i YV has been selected as a robust psychometric approach to evaluate the effectiveness of the programme. In addition, and related with MESSY, Matson et al. (2010) study provides support for the adequate psychometric properties in terms of the construction and validation of this questionnaire. This finding also validates the Spanish version of this instrument (Méndez et al., 2002). Moreover, and related with EQ-i YV, López-Zafra et al. (2014) demonstrated the adequate psychometric properties in terms of the validity of the Spanish version of this questionnaire.

In the same way, the “School Violence in Preschool and Primary School Questionnaire” has been selected because it is an instrument developed in the Spanish education context and is useful for obtaining precise information on different relevant variables such as the type of violence, the places where it occurs, and the frequency of violent behavior. In addition, Albaladejo-Blázquez et al. (2013) showed the adequate psychometric properties in terms of the construction and validation of this questionnaire.

This justifies the adequacy of the choice of instruments such as acute scales to achieve a conceptual adjustment between the instruments and key variables training across the programme, the psychometric indicators for each scale, and the viability for the implementation of this in a sample of young students (8–9 years age range).

In conclusion, based on these criteria, the related instruments have been selected as they are considered appropriate (psychometric consistency and sufficient validity), viable (adequate for the sample and their cultural, education level, and age characteristics) and pertinent for the adjustment of the training on the programme (conflict resolution and promoting democratic co-existence in schools).

### Procedure

The entire school community was fully informed of the details of the study (including the goals, the responsible teacher team, and the confidentiality of the student's answers across all the measure instruments). Prior to participation, written informed consent was obtained. Following this, the participants were randomly assigned to one of the two research conditions: experimental group (where the training on socio-emotional competencies is carried out) or control group (without special training). The experimental group consisted of students who participated in the training programme. The programme was designed to improve their socio-emotional competencies and, in turn, facilitate conflict resolution and promote democratic and peaceful co-existence in schools. The control group consisted of primary school students who did not participate in the programme or receive any other intervention during this period. Finally, the measurement instruments were completed by students before and after the training programme in all research conditions. In order to facilitate the completion of the three questionnaires in such young students, a couple of sessions (of 50 min each) were used, respecting the relevant breaks at all times to avoid students' exhaustion.

### Experimental Design and Data Analysis

A quasi-experimental design “with control group” has been adopted, measuring the variables with the instruments mentioned above following the intervention in both groups (control vs. experimental). Thus, to verify the effectiveness of the training programme implemented, the general linear model (GLM) was implemented. Using this procedure, a multivariate analysis of variance (MANOVA) and a univariate analysis of variance (ANOVA) of repeated measures (factors: group and time) was performed. Tests of within-subjects interaction effects (time × group) were carried out. Finally, the graphs of interactions have been presented to illustrate the differences obtained for both the groups in pretest and posttest settings. SPSS version 22.0 (IBM Corporation, Armonk, NY, United States) has been used for all statistical analyses.

## Results

Firstly, we proceeded to check if the experimental and control groups presented significant differences in the variables considered in our study. For this, a means comparison analysis was carried out for independent samples. On the one hand, and regarding sociodemographic variables, no differences were found between the pre-test and post-test scores between male and female students on any variable. Furthermore, when gender is included as a covariate in the General Linear Model analysis, it is not significant. Therefore, the following statistical analyses were performed by eliminating the gender covariate. On the other hand, the results of student's *t*-test show that there were no significant differences in any of the measured variables between the two groups (experimental vs. control) in pretest, with the exception of the variables “involving violence” and “what you say” (found in the School Violence in Preschool and Primary School Questionnaire), and the Social Skills/Assertiveness (SSA) factor (found in the Matson Evaluation of Social Skills with Youngsters).

Secondly, the M-Box test result indicates the homogeneity of the variance-covariance matrices for the following variables involved in the MESSY: Aggressiveness/Antisocial Behavior (AAB) (*F* = 0.940, *p* = 0.420). This is also applicable for the variables involved on MESSY's Total Scale (*F* = 0.261, *p* = 0.854). Similarly, the following variables were involved in the Emotional Quotient Inventory Short EQ-i YV: intrapersonal intelligence (*F* = 0.432, *p* = 0.430) and stress management (*F* = 1.025, *p* = 0.380). However, the result does not indicate such homogeneity for the following variables involved in the School Violence in Preschool and Primary School Questionnaire: witnessed violence (*F* = 15.198, *p* = 0.000) and lived violence (*F* = 9.984, *p* = 0.000). Further, the following variables were involved in the MESSY: Conceit/Haughtiness (CH) (*F* = 10.910, *p* = 0.000) and Loneliness/Social Anxiety (LSA) (*F* = 5.335, *p* = 0.001). Similarly, the following variables were involved in the Emotional Quotient Inventory Short EQ-i YV: interpersonal intelligence (*F* = 3.356, *p* = 0.018), adaptation (*F* = 4.384, *p* = 0.004), and EQi Total Score (*F* = 0.3.356, *p* = 0.005). In any case, it should be remembered that a violation of this assumption has minimum effect if the groups are approximately equal in size (Hair et al., 1999).

Next, the resulting values of intra-subject and inter-subject effects have been presented in Table 2 to show that the effect of the interaction between the time of evaluation (pretest and posttest) and the implementation of the educational intervention is significant (*p* = < 0.05) for the students involved in the experimental condition in comparison to the control group for the factors found in the School Violence in Preschool and Primary School Questionnaire (witnessed violence, lived violence, involving violence, and “what you see”/“what you say”); for the factors found in the MESSY (Aggressiveness/Antisocial Behavior (AAB), Social Skills/Assertiveness (SSA), Conceit/Haughtiness (CH), Loneliness/Social Anxiety (LSA), and MESSY Total Scale) and all the factors (intrapersonal intelligence, interpersonal intelligence, adaptation and stress management) found in the Emotional Quotient Inventory Short EQ-i YV. So, in all this mentioned factors, the results of the test show that the effect of the interaction between the time of pre-test and post-test assessment and the implementation of the program is significant.

**Table 2:** Results of intrasubjet/intersubject univariate ANOVA.

**Area examined**	**Source**	**Type III**	**df**	***F***	**Sig**.	**Partialη^2^**	**Ob. power**
**School violence**							
**(school violence in preschool and primary school questionnaire)**							
Witnessed violence	Intra	87.49	1.00	21.13	0.00	0.18	1.00
	Intra*Inter	124.45	1.00	30.06	0.00	0.23	1.00
	Error intra	405.73	98.00				
	Inter	31616.33	1.00	2195.03	0.00	0.96	1.00
	Condition	507.45	1.00	35.23	0.00	0.26	1.00
	Error inter	1411.55	98.00				
Lived violence	Intra	0.11	1.00	0.03	0.87	0.00	0.05
	Intra*Inter	106.61	1.00	24.98	0.00	0.20	1.00
	Error intra	418.31	98.00				
	Inter	74352.45	1.00	2864.40	0.00	0.97	1.00
	Condition	240.67	1.00	9.27	0.00	0.09	0.85
	Error inter	2543.83	98.00				
Involving violence	Intra	1.35	1.00	3.32	0.07	0.03	0.44
	Intra*Inter	8.79	1.00	21.60	0.00	0.18	1.00
	Error intra	39.90	98.00				
	Inter	28512.77	1.00	2770.42	0.00	0.97	1.00
	Condition	39.05	1.00	3.79	0.05	0.04	0.49
	Error inter	1008.60	98.00				
“what you see”	Intra	6.86	1.00	9.58	0.00	0.09	0.87
	Intra*Inter	20.90	1.00	29.18	0.00	0.23	1.00
	Error intra	70.19	98.00				
	Inter	29599.48	1.00	2529.21	0.00	0.96	1.00
	Condition	8.48	1.00	0.72	0.40	0.01	0.13
	Error inter	1146.90	98.00				
“what you say”	Intra	0.01	1.00	0.04	0.83	0.00	0.06
	Intra*Inter	7.61	1.00	37.50	0.00	0.28	1.00
	Error intra	19.89	98.00				
	Inter	1380.89	1.00	1226.20	0.00	0.93	1.00
	Condition	3.01	1.00	2.67	0.11	0.03	0.37
	Error inter	110.36	98.00				
**Social skills**							
**[matson evaluation of social skills with youngsters (MESSY)]**							
AAB	Intra	559.73	1.00	235.09	0.00	0.71	1.00
	Intra*Inter	205.53	1.00	86.32	0.00	0.47	1.00
	Error intra	233.33	98.00				
	Inter	355112.36	1.00	1544.65	0.00	0.94	1.00
	Condition	18.88	1.00	0.08	0.78	0.00	0.06
	Error inter	22530.01	98.00				
SSA	Intra	73.96	1.00	55.52	0.00	0.36	1.00
	Intra*Inter	47.18	1.00	35.42	0.00	0.27	1.00
	Error intra	130.54	98.00				
	Inter	2230959.75	1.00	5933.20	0.00	0.98	1.00
	Condition	3271.25	1.00	8.70	0.00	0.08	0.83
	Error inter	36849.25	98.00				
CH	Intra	0.13	1.00	1.59	0.21	0.02	0.24
	Intra*Inter	0.05	1.00	0.64	0.42	0.01	0.12
	Error intra	8.32	98.00				
	Inter	19918.80	1.00	446.68	0.00	0.82	1.00
	Condition	0.40	1.00	0.01	0.92	0.00	0.05
	Error inter	4370.09	98.00				
LSA	Intra	4.12	1.00	5.55	0.02	0.05	0.65
	Intra*Inter	6.92	1.00	9.32	0.00	0.09	0.86
	Error intra	72.77	98.00				
	Inter	48931.18	1.00	3314.58	0.00	0.97	1.00
	Condition	11.98	1.00	0.81	0.37	0.01	0.14
	Error inter	1446.72	98.00				
MESSY total scale	Intra	936.06	1.00	232.05	0.00	0.70	1.00
	Intra*Inter	579.34	1.00	143.62	0.00	0.59	1.00
	Error intra	395.32	98.00				
	Inter	2076317.17	1.00	2563.90	0.00	0.96	1.00
	Condition	4307.89	1.00	5.32	0.02	0.05	0.63
	Error inter	79363.21	98.00				
**Emotional intelligence scores**							
**(emotional quotient inventory)**							
Intrapersonal intelligence	Intra	27.66	1.00	79.11	0.00	0.45	1.00
	Intra*Inter	41.74	1.00	119.38	0.00	0.55	1.00
	Error intra	34.26	98.00				
	Inter	42660.69	1.00	1907.20	0.00	0.95	1.00
	Condition	19.41	1.00	0.87	0.35	0.01	0.15
	Error inter	2192.09	98.00				
Interpersonal intelligence	Intra	8.801	1	17.539	0.000	0.152	0.986
	Intra*Inter	49.301	1	98.245	0.000	0.501	1.000
	Error intra	49.179	98				
	Inter	59312.746	1	3070.190	0.000	0.969	1.000
	Condition	185.526	1	9.603	0.003	0.089	0.866
	Error inter	1893.254	98				
Stress management	Intra	26.79	1.00	21.05	0.00	0.18	1.00
	Intra*Inter	44.59	1.00	35.04	0.00	0.26	1.00
	Error intra	124.71	98.00				
	Inter	54854.25	1.00	4630.07	0.00	0.98	1.00
	Condition	31.81	1.00	2.69	0.10	0.03	0.37
	Error inter	1161.04	98.00				
Adaptation	Intra	17.11	1.00	58.06	0.00	0.37	1.00
	Intra*Inter	15.89	1.00	53.92	0.00	0.35	1.00
	Error intra	28.89	98.00				
	Inter	47267.65	1.00	2513.65	0.00	0.96	1.00
	Condition	6.75	1.00	0.36	0.55	0.00	0.09
	Error inter	1842.83	98.00				
EQ-I total	Intra	5.36	1.00	42.52	0.00	0.30	1.00
	Intra*Inter	16.35	1.00	129.56	0.00	0.57	1.00
	Error intra	12.36	98.00				
	Inter	50817.31	1.00	10022.38	0.00	0.99	1.00
	Condition	27.74	1.00	5.47	0.02	0.05	0.64
	Error inter	496.90	98.00				

**Figure 1:**
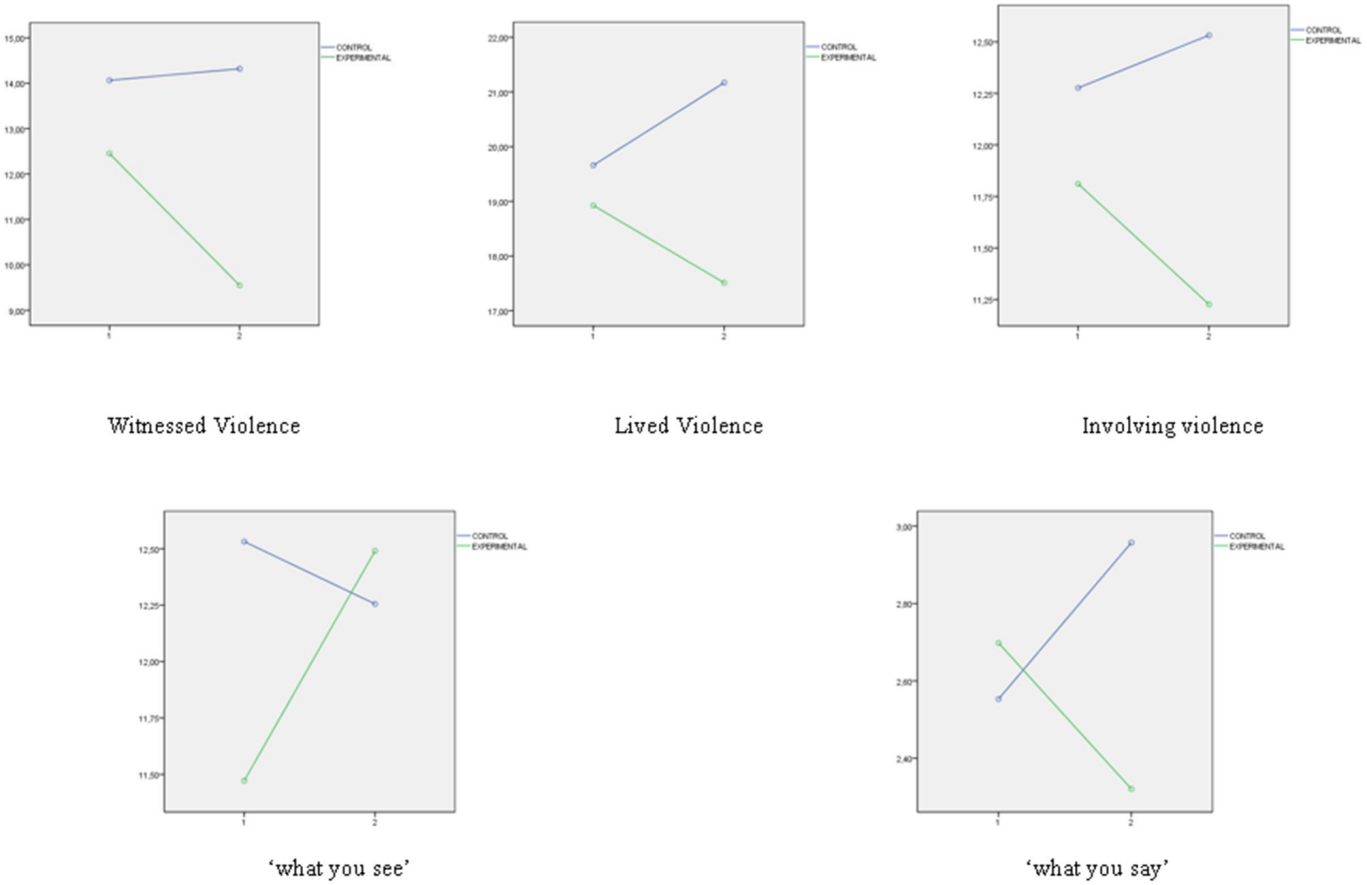
School violence after the educational intervention measure with school violence in preschool and primary school questionnaire.

**Figure 2:**
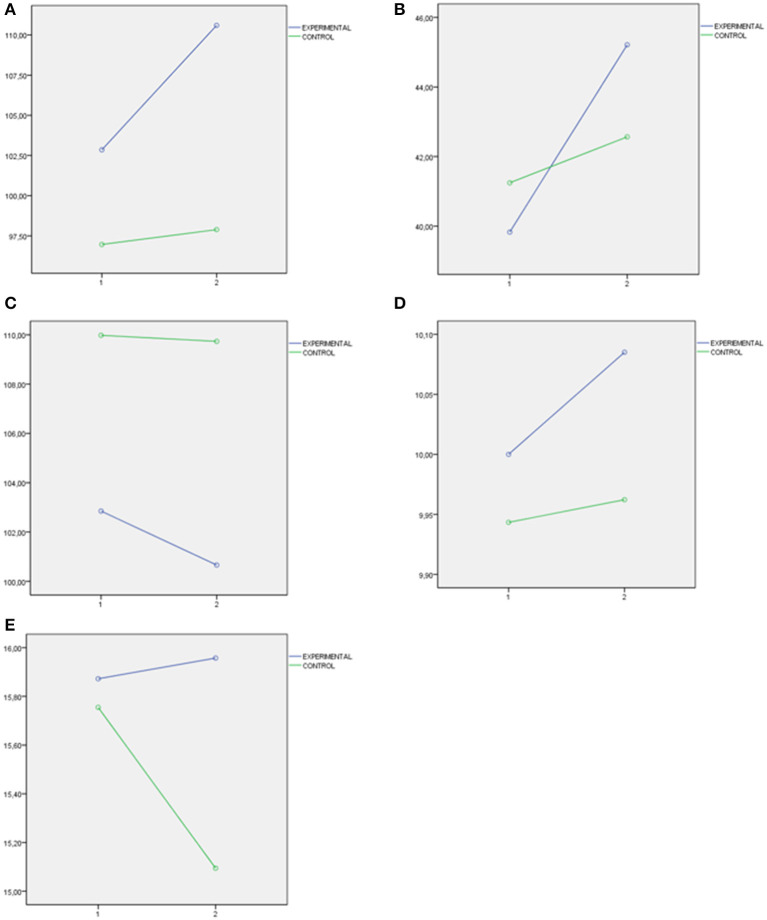
**(A)** MESSY Total Scale as a representation of the significant difference obtained in the set of factor of EI measure with youngsters' social skills measure with Matson Evaluation of Social Skills. **(B)** Aggressiveness/antisocial behavior MESSY. **(C)** Social skills/assertiveness MESSY. **(D)** Conceit/haughtiness MESSY. **(E)** Loneliness/social anxiety MESSY.

**Figure 3:**
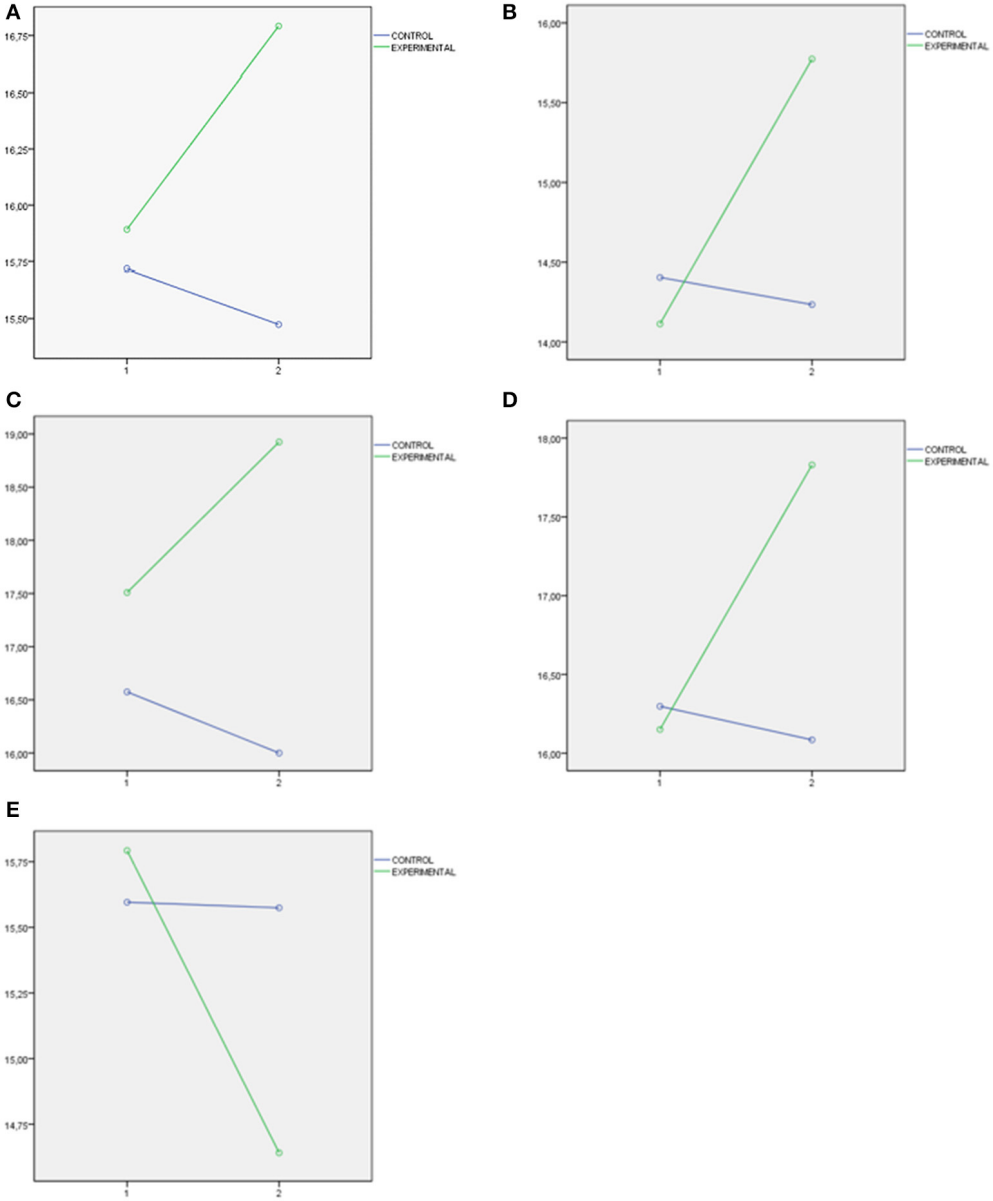
**(A)** Total score EQ-i as a representation of the significant differences obtained in the set of factor of EI measure with Emotional Quotient Inventory Short EQ-i YV. **(B)** Intrapersonal intelligence EQ-i. **(C)** Intrepersonal intelligence EQ-i. **(D)** Stress management EQ-i. **(E)** Adaptation EQ-i.

Finally, three important figures have been presented. Figure 1 presents the interaction graphs that illustrate the directions of the significant differences found in the levels of school violence post the educational intervention (measures with School Violence in Preschool and Primary School Questionnaire). Figure 2 presents the interaction graphs that illustrate the directions of the differences in MESSY Total Score as a representation of the significant differences found in the set of factors of MESSY (Aggressiveness/Antisocial Behavior (AAB), Social Skills/Assertiveness (SSA), Conceit/Haughtiness (CH) and Loneliness/Social Anxiety (LSA). Figure 3 presents the interaction graphs that illustrate the directions of the differences in Total Score EQ-i as a representation of the significant differences found in the set of factors of EI (intrapersonal intelligence, interpersonal intelligence, adaptation and stress management, measures with Emotional Quotient Inventory Short EQ-i YV). Thus, all the factors mentioned showed significant improvements post the intervention for the students involved in the experimental condition.

## Discussion

One of the basic needs of the individual is to feel that he is accepted and appreciated for who he is, to feel that he has an important role within his community, to establish bonds of loyalty, commitment, ethics, and cooperation; as well as obtaining help in times of need, whether on a personal level or as a recipient to overcome the requirements of an academic subject.

For these reasons, it is very important that the curricular designs in the Primary Education stage include teaching strategies, whether based on the master class or mobilized through virtual learning environments, that encourage students to work in unison, develop social skills to handle social situations in the classroom, and appropriately manage social experiences in which an understanding, identification, expression and adequate regulation of own and other emotions is required.

This research is therefore committed to teaching Emotional Intelligence and Social Skills due to their crucial role in the successful prevention of conflicts and in promoting a positive classroom climate and social synergies among Primary Education students.

In recent decades, there has been a rise in the evidences found on the importance of scholar school violence prevention (Gazquez et al., 2007; Loan et al., 2018; Modin et al., 2018; Nation et al., 2020; Xu et al., 2020), the relevance of training Youngsters' Social Skills (Donohue, 2005; Matson et al., 2007; Wang et al., 2011; Youngstrom et al., 2017) and the convenience of instruction for EI competency among the students of primary school (Petrides et al., 2006, 2016; Nelis et al., 2009, 2011; Durlak et al., 2011).

However, while the evidence for the importance of these variables has been highly studied, more front-line applied researches are needed to improve these factors' (prevention of school violence and promotion of socio-emotional skills) importance in the school context in the early academic years (Pozo-Rico and Sandoval, 2020). In the light of the results obtained, the program proposed in this research has been useful for the prevention of violent behaviors in the educational field, promoting the development of social and emotional skills among the students enrolled in the 3rd year of primary education.

On this way, the effectiveness of educational training has been proved for witnessed violence, lived violence, involving violence, and “what you see”/“what you say” found in School Violence in Preschool and Primary School Questionnaire. For the factors Aggressiveness/Antisocial Behavior (AAB), Social Skills/Assertiveness (SSA), Conceit/Haughtiness (CH), Loneliness/Social Anxiety (LSA), and MESSY Total Scale found in the MESSY and all factors (intrapersonal intelligence, interpersonal intelligence, adaptation and stress management) found in Emotional Quotient Inventory Short EQ-i YV. However, higher intervention effect sizes could have been obtained with a larger sample of students. In any case, all this mentioned factors, the results of the test show that the effect of the interaction between the time of pre-test and post-test assessment and the implementation of the program is significant.

Note that the type of activity the control group had instead of the treatment group is the regular academic classroom in the context of the normative and standard curriculum. The experimental group consisted of students who participated in the training programme. The programme was designed to improve their socio-emotional competencies and, in turn, facilitate conflict resolution and promote democratic and peaceful co-existence in schools. The control group consisted of primary school students who did not participate in the programme or receive any other special co-existence intervention during this period, but continued with the regular academic classroom instead. Hence, the convenient regular academic activity related with the standard curriculum in the control group could not have detrimentally affected involved groups of children. In neither case this control students group has been unobserved in view of the fact that they have the regular classes based on the academic curriculum all the school time.

This study therefore presents an opportunity to impart socio-emotional competencies among primary school students to improve conflict resolution and promote democratic and peaceful co-existence in schools. In addition, an enriched version of educational training is highly applicable to other educational contexts to enhance students' personal development and decrease the levels of violence found in primary education.

## Conclusion

To summarize, the study theoretically implies that there should be a promotion of socio-emotional skills and prevention of school violence among primary education students *via* committed and effective educational programs, such as the one shown in this research. The study's practical implications are based on the fact that the proposed program is a simple and an easily applicable intervention which any teacher—who is trained to teach its contents—can put into practice in his/her classroom.

However, there is a need for future research to address the research gaps of this study along seven fundamental lines: (1) replicate the study with a larger sample size to ensure that the research is carried out with the groups of each comparable experimental condition and ensure that there are no significant differences between any of the variables studied; (2) include more training regarding the violence committed and haughtiness (related to social skills) in the student training program to facilitate significant differences between the factors; (3) expand the evaluation of the prevention of school violence and the promotion of social skills among teachers and families (for the instruments used in this study allow it), as well as introduce a new instrument for the evaluation of emotional intelligence valued by the two key educational agents (teachers and families); (4) conduct a long-term educational follow-up to evaluate if the positive results of the intervention are maintained over time; (5) it would be interesting to apply the program to the different stages of all the courses and adapt it for different ages, thereby obtaining better results at the school level; (6) it would be convenient to design a training program for teachers and families who complete the action with students; (7) include a greater number of variables in future research to enable the evaluation of the other beneficial aspects of the program and its impact on key issues, such as academic performance and well-being of students (and the entire educational community); and (8) New instruments must be added to cover all the content of the programme in a more exhaustive way. These new lines of research to prevent school violence and promote socio-emotional skills among students are evidenced as attractive and committed to facilitating quality teaching and facing the new educational challenges of the 21st century.

In conclusion, violence is extremely common in modern society. It is present in families, schools, and the media. Promoting co-existence in classrooms has become a priority objective that does not always achieve the desired effects.

Numerous studies focus their efforts on generating proposals that provide solutions to problems related to violence against others, and also against oneself. The scientific literature presented a multitude of programmes aimed at eradicating violence or bullying in the classroom, eliminating gender-based violence, and developing an endless number of projects that seek to appease a series of problems that often have a common origin: deficiencies in students' socio-emotional skills. In this way, meta-studies of Durlak (2015a), Durlak (2015b), and Taylor et al. (2017) are especially relevant because they provide evidence of the importance and consequences of EI and social skills improvement (as obtained in the current study) to derive a positive impact on the wider research and policy context more concerned with democratic co-existence in schools.

In this way, through the training proposed herein, this research generates a socio-affective environment in which students can achieve full personal and social development. In addition, a large part of the development of a child's personality takes place during primary school, the educational environment in which the most important social interactions will take place.

For this reason, studies on violence in childhood should focus on the educational context so that, together with the participation of families, a climate that favors co-existence can be achieved. We can affirm that by promoting the socio-emotional skills of our students, we have managed to prevent the appearance of violent behaviors in the classroom. So, the way educational systems are organized to incorporate these types of programmes is key to guaranteeing the same results as obtained in our research and reducing instances of school violence.

Therefore, the results clearly indicate it is possible to achieve an improvement in social skills and the management of emotions in the improvement of conflict resolution and the promotion of democratic coexistence in schools. This has great practical implications for achieving the state of well-being and quality education to which we aspire in the educational field.

## Data Availability Statement

The raw data supporting the conclusions of this article will be made available by the authors, without undue reservation.

## Ethics Statement

The studies involving human participants were reviewed and approved by Ref. UA2015-07-06. Written informed consent to participate in this study was provided by the participants' legal guardian/next of kin.

## Author Contributions

MS-V, TP-R, and RG-C: conceptualization. JC: methodology. RG-C: software and data curation. TP-R and RG-C: validation. JC and RG-C: formal analysis. RG-C, TP-R, JC, and MS-V: writing—original draft preparation. RG-C, TP-R, and JC: writing—review and editing. All authors have read the manuscript and have agreed to have a published version of the manuscript.

## Conflict of Interest

The authors declare that the research was conducted in the absence of any commercial or financial relationships that could be construed as a potential conflict of interest.
